# Differential Ratios of Omega Fatty Acids (AA/EPA+DHA) Modulate Growth, Lipid Peroxidation and Expression of Tumor Regulatory MARBPs in Breast Cancer Cell Lines MCF7 and MDA-MB-231

**DOI:** 10.1371/journal.pone.0136542

**Published:** 2015-09-01

**Authors:** Prakash P. Mansara, Rashmi A. Deshpande, Milind M. Vaidya, Ruchika Kaul-Ghanekar

**Affiliations:** 1 Cell and Translational Research Lab, Interactive Research School for Health Affairs (IRSHA), Bharati Vidyapeeth University Medical College Campus, Dhankawadi, Pune, 411043, India; 2 Advanced Centre for Treatment, Research & Education in Cancer (ACTREC), Tata Memorial Centre, Kharghar, Navi Mumbai, 410210, India; Fundação Oswaldo Cruz, BRAZIL

## Abstract

Omega 3 (n3) and Omega 6 (n6) polyunsaturated fatty acids (PUFAs) have been reported to exhibit opposing roles in cancer progression. Our objective was to determine whether different ratios of n6/n3 (AA/EPA+DHA) FAs could modulate the cell viability, lipid peroxidation, total cellular fatty acid composition and expression of tumor regulatory Matrix Attachment Region binding proteins (MARBPs) in breast cancer cell lines and in non-cancerous, MCF10A cells. Low ratios of n6/n3 (1:2.5, 1:4, 1:5, 1:10) FA decreased the viability and growth of MDA-MB-231 and MCF7 significantly compared to the non-cancerous cells (MCF10A). Contrarily, higher n6/n3 FA (2.5:1, 4:1, 5:1, 10:1) decreased the survival of both the cancerous and non-cancerous cell types. Lower ratios of n6/n3 selectively induced LPO in the breast cancer cells whereas the higher ratios induced in both cancerous and non-cancerous cell types. Interestingly, compared to higher n6/n3 FA ratios, lower ratios increased the expression of tumor suppressor MARBP, SMAR1 and decreased the expression of tumor activator Cux/CDP in both breast cancer and non-cancerous, MCF10A cells. Low n6/n3 FAs significantly increased SMAR1 expression which resulted into activation of p21^WAF1/CIP1^ in MDA-MB-231 and MCF7, the increase being ratio dependent in MDA-MB-231. These results suggest that increased intake of n3 fatty acids in our diet could help both in the prevention as well as management of breast cancer.

## Introduction

Breast cancer is the most common malignancy and one of the leading cause of cancer-related deaths in women worldwide [1, 2]. Several factors have shown promise in reducing breast cancer incidence rates wherein change in lifestyle, especially diet, has proven to be the most popular measure. The role of nutrition in the prevention of cancer has been well established and it has been shown to suppress the transformative, hyper-proliferative and inflammatory processes that initiate carcinogenesis [3].

During the past few years, there has been a wealth of information regarding the role of long chain polyunsaturated fatty acids (LCPUFAs) in health and disease [4–7]. n3 FA such as ALA (Alpha-linolenic acid) [8], EPA (Eicosapentaenoic acid) [9] and DHA (Docosahexaenoic acid) [10] have been reported to exhibit anti-cancer activity whereas n6 PUFAs such as linoleic acid (LA) and arachidonic acid (AA)[11–13] have been reported to contribute towards the development of cancer. EPA and DHA are essential fatty acids, which human body cannot synthesize and thus should be obtained from diet. AA, EPA and DHA occur in the diet in animal tissue lipids [14]. Fish oil is highly rich in EPA and DHA, and has been suggested for different populations due to health benefits [15]. EPA and DHA together have been recommended in various conditions such as coronary, CVD, CHD, Alzheimer, postpartum depression and bipolar depression, rheumatoid arthritis, pregnancy, lactation and infancy and even cancer [15]. In our recent study, we found that supplementation of fish oil capsules, containing EPA:DHA in the ratio of 1.5:1, in breast cancer patients undergoing chemotherapy, significantly improved their serum antioxidant levels as well as quality of life parameters [16].

Various mechanisms have been proposed for the anti-proliferative effect of n-3 PUFAs [17]. These include alterations in eicosanoid formation [18], lipid peroxidation initiated by free radicals [8, 19], accumulation of cytotoxic lipid droplets [20], and specific changes in gene expression patterns [8, 17]. Recently, we have reported that ALA regulated the growth of breast and cervical cancer cells through decrease in NO generation and increase in LPO, leading to caspase 3-dependent apoptosis [8]. The activity of several nuclear transcription factors, like peroxisome proliferator-activated receptors (PPARα/δ/γ), liver X receptors (LXRα/β), and sterol regulatory element-binding proteins (SREBP1/2), has been shown to be regulated by dietary PUFAs and their metabolites [21–23]. Moreover, tumor suppressor proteins such as p53 [24, 25], BRCA1 [26], BRCA2 [26], syndecan-1 (SDC-1) [22] as well as PTEN [27] have also been reported to be upregulated in cells challenged with n3 fatty acids.

Several studies have reported an inverse correlation between the ratios of n6/n3 fatty acids (FAs) and the risk of developing breast cancer [17, 28, 29]. The consumption of n6 PUFAs has considerably increased in the recent times. The current western diet has n6/n3 ratio ranging from 20-25/1 compared to the ratio of 1/1 that was prevalent in the diet of our ancestors [30]. High n6/n3 ratios favor the formation of pro-inflammatory eicosanoids from LA [31] that leads to the development of various disorders including cancer [32]. In vivo studies using corn oil (n6 FA) and its different ratios with fish oil (n3 FA) (n6/n3 ratio: 1/1, 1/1.5/1/9) [33, 34] have established the antineoplastic potential of n3 PUFAs in breast cancer as well as in colon cancer (n6/n3 ratio: 1/1, 1/2.5) [35, 36]. Few other studies as reviewed in [37] have reported protective effects of varying n6/n3 ratios in breast cancer. However, to our knowledge, the effect of equal (1/1), low (1/2.5, 1/4, 1/5, 1/10) and high (2.5/1, 4/1, 5/1, 10/1) ratios of n6/n3 PUFAs on cell viability, lipid peroxidation and total cellular fatty acid composition have not been studied in detail in breast cancer cell lines. In addition, we are for the first time reporting the modulation of tumor regulatory MARBPs (nuclear matrix associated Matrix Attachment Region binding proteins) such as SMAR1 (scaffold/matrix attachment region binding protein 1) and Cux/CDP(CCAAT-displacement protein/cut homeobox), in response to different n6/n3 FAs. Interestingly, the expression of cell cycle regulatory protein p21^WAF1/CIP1^ was modulated in MDA-MB-231 and MCF7 cells treated with different n6/n3 FA ratios. This is the only study in vitro showing the effect of high and low ratios of n6/n3 FAs on cellular mechanisms in cancerous and non-cancerous cells.

## Materials and Methods

### Materials

Tissue culture plastic ware was purchased from BD Bio-sciences (CA, USA). Dulbecco's Modified Eagles Medium (DMEM), penicillin and streptomycin were obtained from Gibco BRL (CA, USA). Fetal bovine serum (FBS) was purchased from SAF (USA). Bovine serum albumin (BSA) fatty acid free, 3-(4,5-Dimethylthiazol-2-yl)-2,5-diphenyl tetrazolium bromide (MTT), Epidermal Growth Factor(EGF), Insulin, Hydrocortisone and the other reagents were obtained from Sigma-Aldrich (St. Louis, MO, USA). EPA was purchased from MERCK whereas DHA and AA were purchased from Cayman. α-Tubulin, p21^WAF1/CIP1^ and Cux/CDP antibodies were obtained from Santa Cruz Biotechnology Inc.(CA, USA). SMAR1 antibody was purchased from Bethyl laboratories, Montgomery, USA. All other common reagents were procured from Qualigens Fine chemicals and Himedia (Mumbai, India).

### Cell culture

The human breast adenocarcinoma (MCF7 and MDA-MB-231) cell lines used in the study were obtained from National Centre for Cell Science (NCCS), Pune, India. The cells were grown in DMEM containing 2 mM L-glutamine supplemented with 10% fetal bovine serum (FBS), 20 U/ml penicillin and 20 μg/ml streptomycin [8]. Immortalized non-tumourigenic human breast epithelial cells, MCF10A was cultured under standard conditions in 1/1 (v/v) mixture of DMEM and Ham’s F12 medium (DMEM/F12) containing 2 mM L-glutamine, 10% FBS, 20 U/ml penicillin, 20 μg/ml streptomycin, 10 μg/ml insulin, 20 ng/ml EGF and 0.5 mg/ml hydrocortisone [38]. The cells were incubated in a humidified 5% CO_2_ incubator at 37°C.

### Conjugation of BSA with Omega Fatty Acids

Eicosapentaenoic acid (EPA), Docosahexanoic acid (DHA) and Arachidonic acid (AA) were dissolved in absolute ethanol and stored at -20°C. The fatty acids were conjugated with delipidated, endotoxin-free serum albumin (3mM) to give a concentration of 10 mM stock with a ratio of fatty acids to BSA as 3/1 [8, 39]. The conjugated omega fatty acids were incubated at 37°C for 30 min in CO_2_ incubator and stored at -20°C and before use; they were diluted to the required concentration with 10% DMEM.

### Cell viability study by MTT dye reduction assay

The cell viability was determined by MTT assay in breast cancer cell lines (MCF7 and MDA-MB-231) in presence of different concentrations of n3 and n6 fatty acids and compared with non-cancerous immortalized MCF10A cell lines. The cells were seeded at a density of 2×10^4^ cells/well density in 96-well plates and grown for 24 h. The cells were treated with different ratios (1/1, 2.5/1, 4/1, 50 and 10/1) of n3 (EPA and DHA) and n6 (AA) fatty acids. Table 1 shows the various ratios of fatty acids that were used in the experiment. After 24h, media was removed, MTT solution (5 mg/ml) was added to each well and the cells were cultured for another 4 h at 37°C in 5% CO_2_ incubator. The formazan crystals formed were dissolved in 90 μl of SDS-DMF (20% SDS in 50% DMF)[40]. After 15 min, the amount of colored formazan derivative was determined by measuring optical density (OD) at 570 nm (OD 570–630 nm) using BMG FLUOstar Omega microplate reader.

**Table 1:** Concentrations of n6 (AA) and n3 (EPA, DHA) fatty acids used in different ratios at 200 μM total fatty acid concentration.

		Low n6/n3 Ratios				High n6/n3 Ratios			
Fatty acids	1:1	1:2.5	1:4	1:5	1:10	2.5:1	4:1	5:1	10:1
**AA (μM)**	100.00	57.14	40.00	33.33	18.18	142.86	160.00	166.67	181.82
**EPA (μM)**	60.00	85.71	96.00	100.00	109.09	34.29	24.00	20.00	10.91
**DHA (μM)**	40.00	57.14	64.00	66.67	72.73	22.86	16.00	13.33	7.27

### Cell growth analysis

The assay was performed as described previously [22]. Briefly, MCF7, MDA-MB-231 and MCF10A cells were seeded at a density of 2×10^4^ cells/well in 24-well plates in triplicates. Next day, the cells were treated with different concentrations of n3 and n6 FA ratios for 24 h as shown in Table 2. The cells were harvested and counted for viability using trypan blue dye exclusion method.

**Table 2:** Concentrations of n6 (AA) and n3 (EPA, DHA) fatty acids used in different ratios at 280 μM total fatty acid concentration.

		Low n6/n3 Ratios				High n6/n3 Ratios			
Fatty acids	1:1	1:2.5	1:4	1:5	1:10	2.5:1	4:1	5:1	10:1
**AA (μM)**	140.00	80.00	56.00	46.67	25.45	200.00	224.00	233.33	254.55
**EPA (μM)**	84.00	120.00	134.40	140.00	152.73	48.00	33.60	28.00	15.27
**DHA (μM)**	56.00	80.00	89.60	93.33	101.82	32.00	22.40	18.67	10.18

### Lipid peroxidation assay

All the cells were seeded at density 2×10^4^ cells/well in a black 96-well plate and incubated at 37°C in CO_2_ incubator. Next day, the cells were treated with various n3 and n6 FA ratios and incubated in CO_2_ incubator at 37°C for 24 h. The following day, the medium was removed and the cells were washed with 1X PBS and incubated with fluorescent indicator, cis-parinaric acid (CPNA) (Invitrogen) as described previously [41]. Briefly, the cells were incubated with 10μM cis-parinaric acid and incubated for 60 min at 37°C in dark. 20μM tert-butyl hydroperoxide (TBHP) was kept as a positive control and was incubated with CPNA dye. After incubation, cells were washed twice with 1X PBS. 100μl 1X PBS was added to each well, and the fluorescence readings were immediately taken on FLUOstar Omega-Multi-mode micro plate reader (BMG LABTECH) with maximum excitation and emission wave length of 320 nm and 420 nm, respectively. Decreased fluorescence reflects increased lipid peroxidation.

### Cellular fatty acid analysis

Breast cancer cell lines were plated at 8×10^5^ cells/well density in 6 well plates and were incubated in 10% DMEM supplemented with n3 and n6 fatty acids for 24 h. The procedure for fatty acid analysis used in our study was adapted from the Manku et al [42]. Trans-esterification of total cellular lipid fractions was carried out with hydrochloric acid-methanol using a Perkin Elmer gas 118 chromatograph (SP 2330, 30m capillary column, Supelco, PA, USA). Briefly, cell pellets were dissolved in 500 μL 1X PBS and mixed with 4 ml of Methanol/HCl/BHT (94.7/5.3/0.0005, v/v/w) in a 15-ml screw cap vial. The vials were sealed and incubated at 80°C for 2 h and then cooled on ice for 30 min. The total methylated fatty acids were extracted by adding 2 ml hexane (2N), and the layers were separated by centrifugation in a swinging rotor at 3000 rpm for 15 min at room temperature. The hexane layers were carefully removed and collected in a separate vial. The hexane extract was completely dried by passing argon gas and stored at -20°C until analyzed. The methylated fatty acids were re-suspended in 100μL of chloroform, and 1μL was injected in GC column. Helium was used as carrier gas at 1 ml/min. Oven temperature was held at 150°C for 10 min, programmed to rise from 150 to 220°C at10°C/min, and at 220°C for 10 min. The detector temperature was 275°C, and the injector temperature was 240°C. The column was calibrated by injecting the standard fatty acid mixture in approximately equal proportion. The data was recorded and the peaks were identified as per the retention time of the standard fatty acids (Sigma, USA) run under the identical conditions. Individual fatty acids were expressed as a relative percentage of total analyzed fatty acids.

### Western blotting

Total cellular protein isolation and western blotting were performed as previously described by Wani et al [40]. Cell extracts were prepared from control (untreated) as well as cells treated with different n3 and n6 ratios (1/1, 2.5/1, 4/1, 5/1 and 10/1). Briefly, the cell pellet was resuspended in 50 μl lysis buffer containing 50 mM Tris (pH 7.4), 5 mM EDTA, 0.5% NP40, 50 mM NaF, 1 mM DTT, 0.1 mM PMSF, 0.5 μg/ml leupeptin, 1 μg/ml pepstatin, 150 mM NaCl, 0.5μg/ml aprotinin and protease inhibitor cocktail and incubated on ice for 1 h with intermittent mixing. The protein extract was centrifuged for 20 min at 4°C at 12,000 rpm. The protein was estimated by using Bradford reagent (Biorad Laboratories Inc, CA, USA). Equal amount of protein was loaded onto a 10% SDS-polyacrylamide gel and transferred to Amersham Hybond-P PVDF membrane (GE Healthcare, UK). The membrane was blocked in 10% BSA in Tris-buffered saline containing 0.1% Tween-20 (TBST) and incubated at room temperature for 4 h. Primary antibodies such as mouse monoclonal antibody alpha-tubulin (sc-5286, 1:500 dilution), p21 (sc-817, 1:500 dilution); rabbit polyclonal SMAR1 (A300-279A, 1:1000 dilution) and Cux/CDP (sc-13024, 1:1000 dilution) were incubated with the membrane for 4 h. The membrane was washed in TBST and incubated with either donkey anti-mouse IgG-HRP conjugated (for alpha-tubulin, p21) or donkey anti-rabbit IgG-HRP conjugated (for SMAR1 and Cux/CDP). Proteins were visualized using a chemiluminescence kit (Amersham ECL Advance western blotting detection kit, GE Healthcare, UK) and densitometry analysis of X-ray films was performed on ImageJ software (Image Processing and Analysis in Java; NIH, Bethesda, MD, USA).

### Data analysis

The data analysis was done by using GraphPad prism 5 (San Diego, USA). The results have been presented as mean±SEM. Data was analyzed by using analysis of variance (ANOVA) test to compare the means. A significant F test was followed by post hoc Turkey’s multiple comparison test. Kruskal–Wallis test was used wherever the homogeneity of variance test failed. For the comparisons between effect of low and high n6/n3 FAs, data was analyzed by unpaired student’s t-test. The level of significance used was p<0.05.

## Results

### Differential ratios of n6 and n3 FA regulated the viability and proliferation of breast cancer cells

We first analyzed the effect of low and high ratios of n6 and n3 fatty acids (AA/EPA+DHA) on the regulation of viability and growth of breast cancer cell lines, MDA-MB-231 and MCF7.

**Fig 1:**
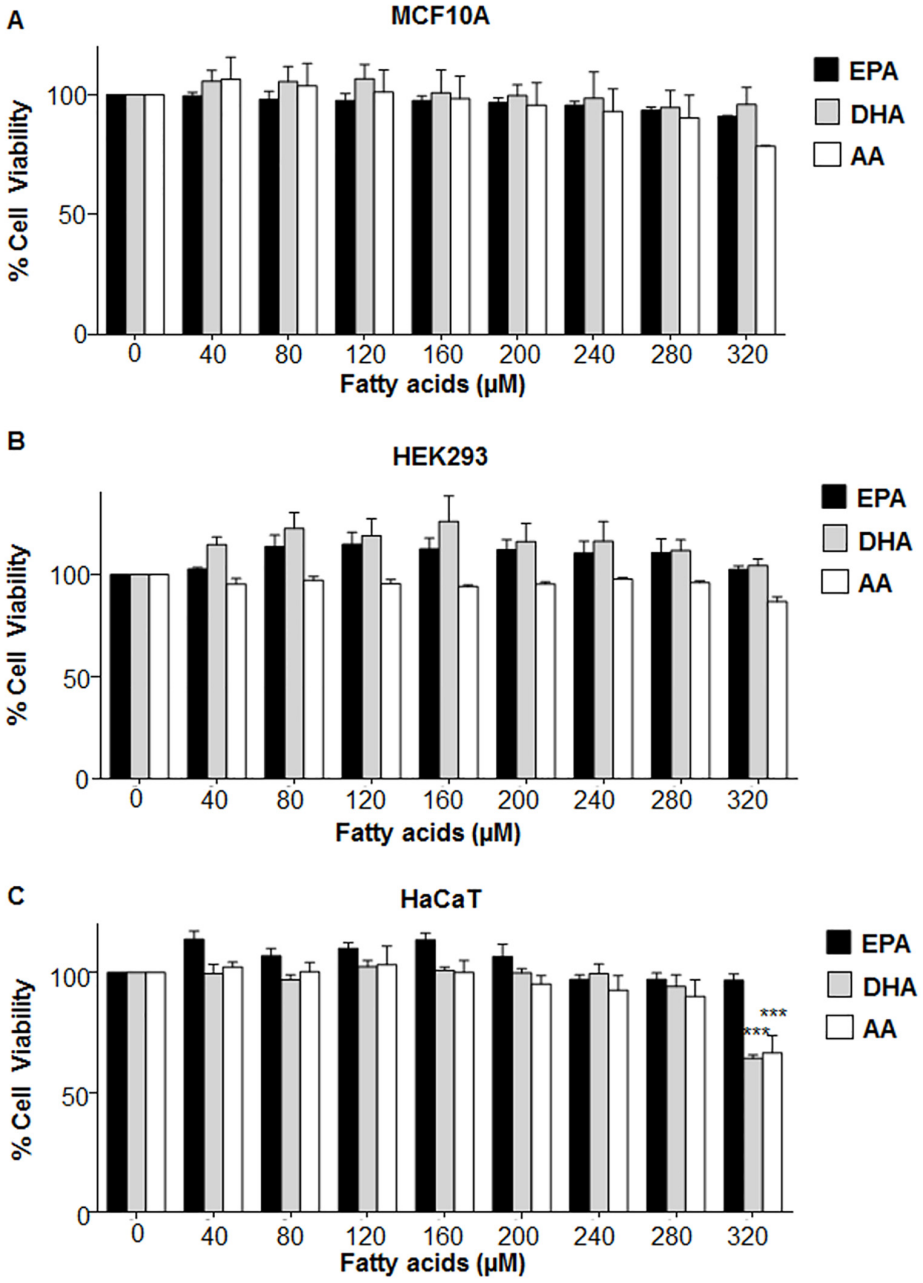
Effect of different concentrations of EPA, DHA and AA on viability of non-cancerous transformed cells. (A) MCF10A, (B) HEK293 and (C) HaCaT cells were exposed to different concentrations (0–320μM) of either EPA, DHA, AA and MTT assay was performed. Data has been presented as mean±SEM of three independent experiments, performed in 96 well plates. Statistical significance was assayed by one-way ANOVA, followed by a Dunnett's test. ***p<0.01.

**Fig 2:**
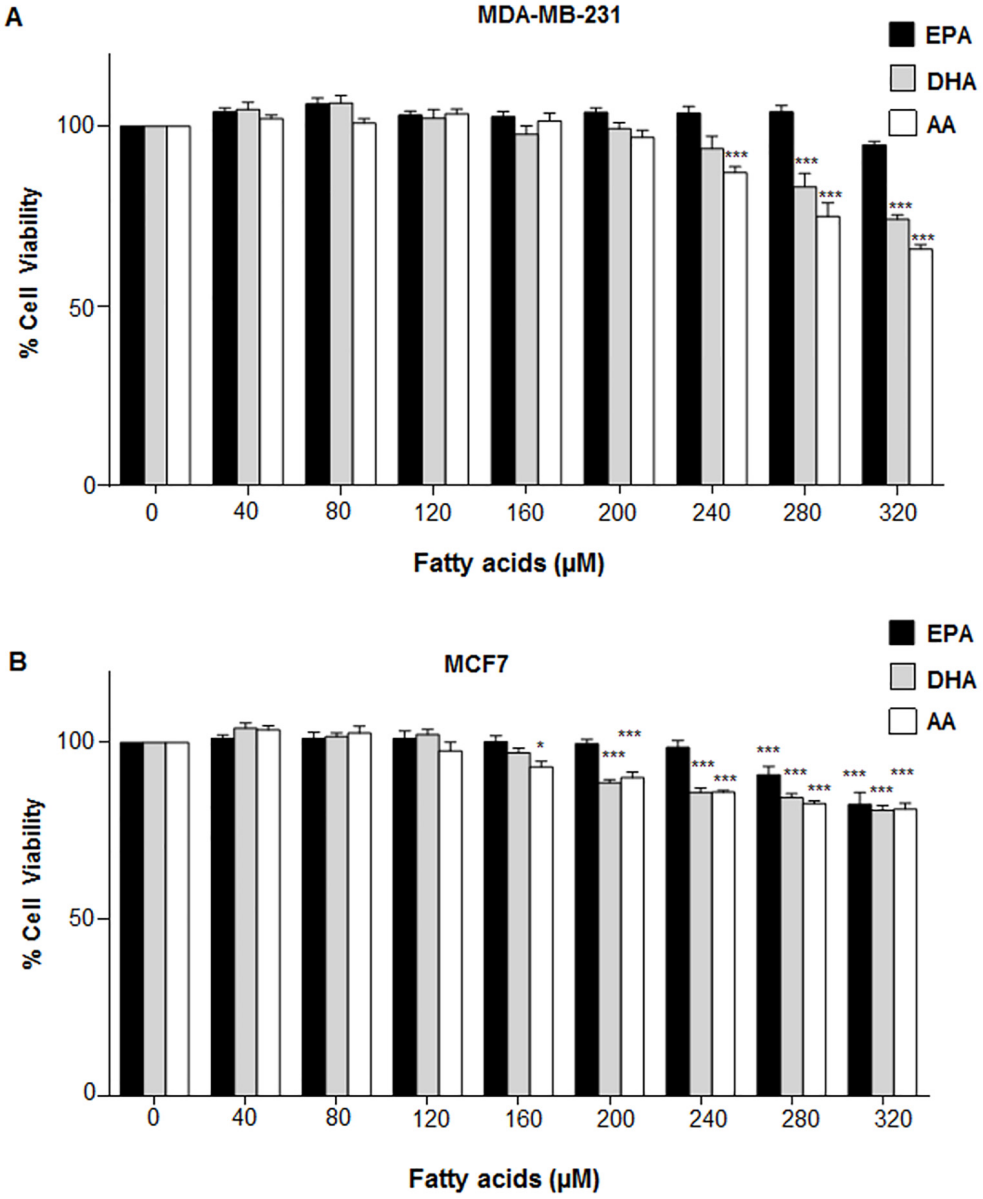
Effect of different concentrations of EPA, DHA and AA on viability of breast cancer cells. (A) MDA-MB-231 and (B) MCF7 cells were treated with different concentration of EPA, DHA and AA for 24 h, followed by cell viability analysis by MTT assay. Data has been presented as mean±SEM of three independent experiments, each performed in 96 well plates. Statistical significance was assayed using one-way ANOVA, followed by a Dunnett's test.*p<0.05, ***p<0.001.

The study was preceded by following preliminary tests to determine the appropriate concentrations of individual EPA, DHA and AA that would be used in different n6/n3 ratios. It was found that upto 280 μM concentration, the individual fatty acids (EPA, DHA and AA) were almost non-toxic to MCF10A, HEK 293 and HaCaT (Fig 1); as well as MDA-MB-231 and MCF7 (Fig 2).

**Fig 3:**
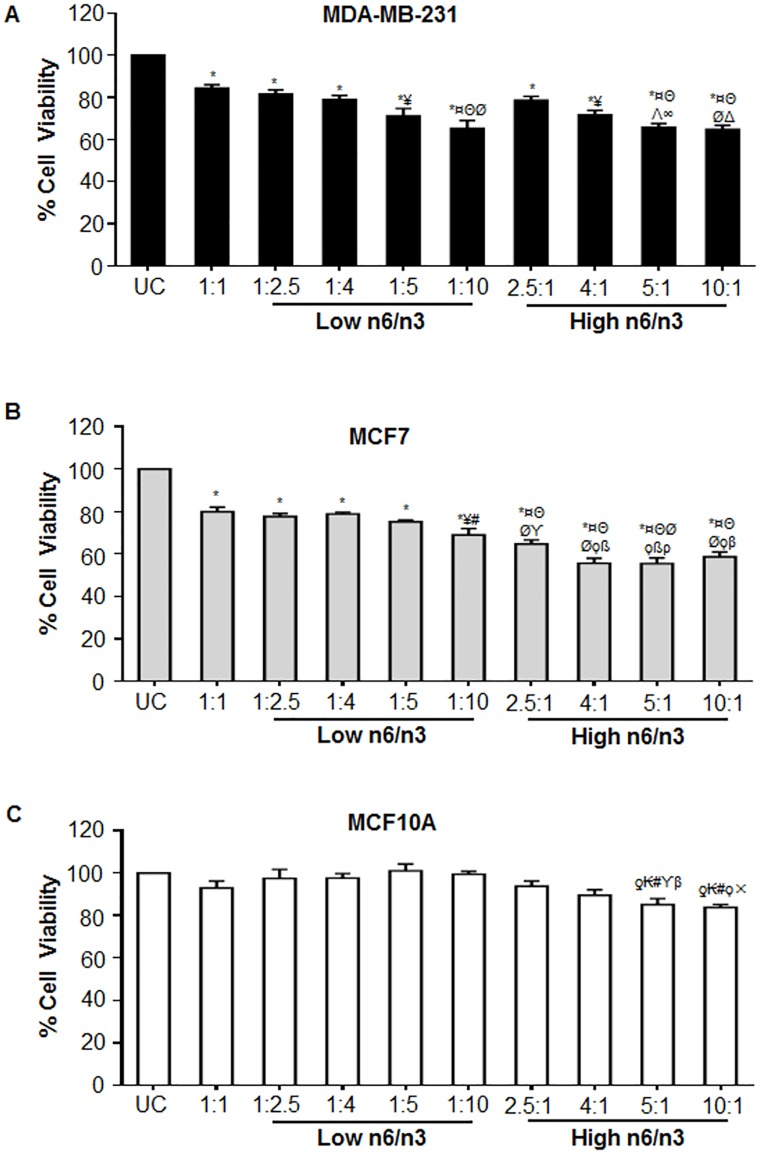
Differential ratios of n6 and n3 fatty acids regulate the viability of breast cancer and non-cancerous cells. Breast cancer cell lines, (A) MDA-MB-231 and (B) MCF7 as well as immortalized non-tumorigenic human breast epithelial, MCF10A cells (C) were treated with low and high n6/n3 ratios and analyzed for cell viability by MTT assay. Data has been presented as mean±SEM of five independent experiments, each conducted in triplicates. ƍp<0.01 and *p<0.001 compared to UC; ¥p<0.01 and ¤p<0.001 compared to 1:1; Θp<0.001 compared to 1:2.5; #p<0.05, Λp<0.01 and Øp<0.001 compared to 1:4;ϒp<0.01 and ϙp<0.001 compared to 1:5; βp<0.05, ×p<0.01 and ßp<0.001 compared to 1:10; ∞p<0.01 and Δp<0.001 compared to 2.5:1.

To determine the effective ratios of AA/EPA+DHA that would decrease cell viability, we tested two different concentration, 200 and 280 μM, of total fatty acids wherein the ratio of n6 (AA) and n3 (EPA and DHA) FAs that was used has been mentioned in Tables 1 and 2, respectively. We initially kept the total FA concentration (of each n6/n3 ratio) at 200 μM (Table 1) and treated the cells with different ratios. After performing MTT assay, we didn’t find any effect on cell viability of MDA-MB231 and MCF7 (Table 3). So, we increased the total FA concentration to 280 μM (Table 2), which was found to affect the cell viability of breast cancer cells significantly (Fig 3) and thus further experiments were performed with 280 μM concentrations of total FAs. In the experiments, EPA:DHA was maintained at 1.5:1 ratio based on the composition of standard commercial fish oil supplements [15] as well as our recent report[16].

**Table 3:** Effect of differential ratios of n6 (AA) and n3 (EPA+DHA) fatty acids on breast cancer cell viability at 200 μM total FA concentration.

		MDA-MB-231	MCF7
**Untreated Control (UC)**		100.00±0.00	100.00±0.00
	**1:1**	100.20±1.59	101.60±4.28
**Low n6/n3 FA ratios**	**1:2.5**	104.00±2.00	98.03±4.80
	**1:4**	102.20±1.92	97.37±1.26
	**1:5**	104.60±2.69	92.02±2.37
	**1:10**	104.40±2.24	92.57±1.38
**High n6/n3 FA ratios**	**2.5:1**	103.00±4.10	99.20±2.02
	**4:1**	103.90±3.25	96.66±2.86
	**5:1**	99.08±1.96	94.79±2.95
	**10:1**	102.20±2.49	93.37±3.15

It was observed that at equal ratio (1:1) of n6/n3 fatty acids, MDA-MB-231, MCF7 and MCF10A showed upto 84, 80 and 92% survival (p<0.05) compared to the untreated control (UC) cells, respectively (Fig 3). However, with decreasing n6/n3 FA ratios, a significant ratio-dependent decrease in cell survival of only breast cancer cell lines (MDA-MB-231 and MCF7) (Fig 3A and 3B) was observed, with almost no reduction in cell viability of MCF10A (p<0.05) (Fig 3C). It was observed that compared to equal ratio (1:1) of n6/n3, 1:10 ratio showed significant decrease in the viability of breast cancer cells, MDA-MB-231 (p<0.001) and MCF7 (p<0.01). On the other hand, with increasing ratios of n6/n3fatty acids, a ratio-dependent decrease in the cell survival was observed not only in the breast cancer (Fig 3A and 3B) but also in MCF10A (Fig 3C). Similar results were obtained in non-cancerous transformed cell lines, HaCaT and HEK293, wherein low n6/n3 did not affect their viability compared to higher ratios (S1 Fig).

**Fig 4:**
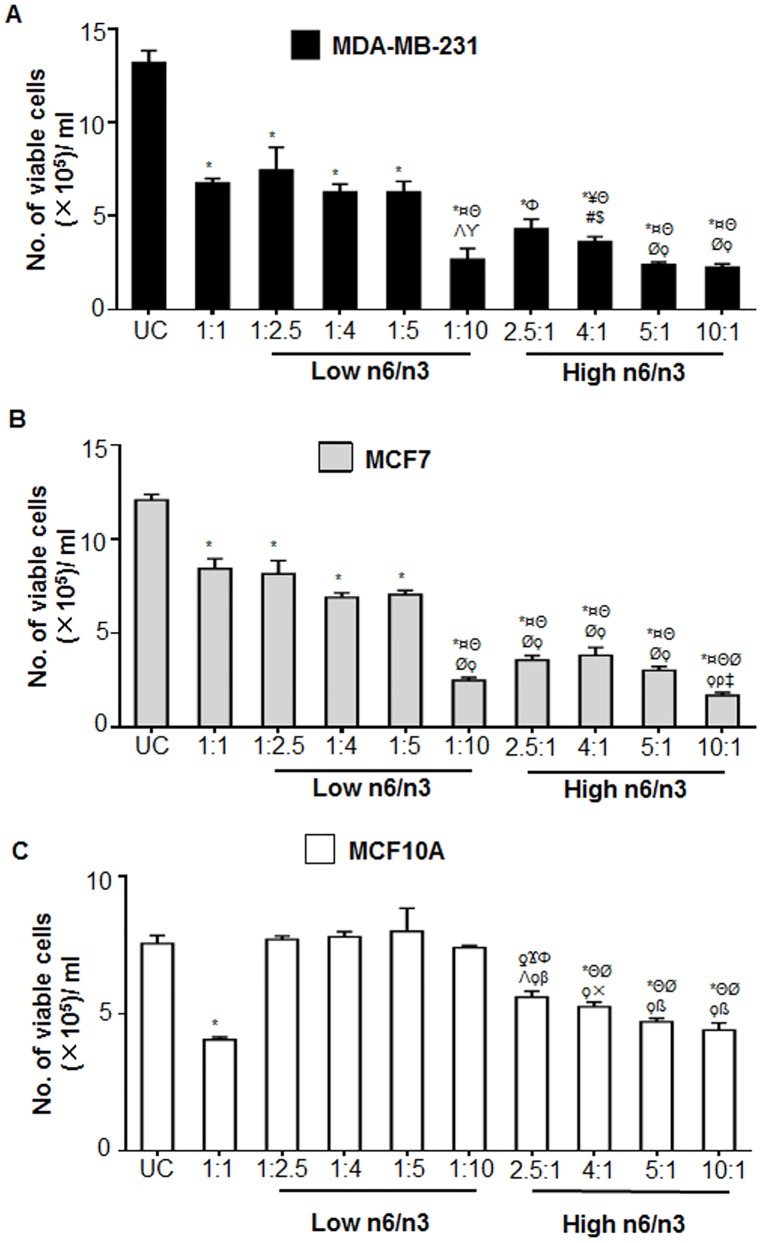
Various ratios of n6 and n3 regulate the cell proliferation of breast cancer and non-cancerous cell lines. MDA-MB-231(A), MCF7 (B) as well as MCF10A (C) cell lines were treated with low and high n6/n3 ratios for 24h. Next day, the number of viable cells was counted using the trypan blue dye exclusion assay. Data has been presented as mean±SEM of three independent experiments, each conducted in triplicates. ƍp<0.01 and *p<0.001 compared to UC; Ϫp<0.05, ¥p<0.01 and ¤p<0.001 compared to 1:1; Φp<0.01 and Θp<0.001 compared to 1:2.5; #p<0.05, Λp<0.01 and Øp<0.001 compared to 1:4; $p<0.05, ϒp<0.01 and ϙp<0.001 compared to 1:5; βp<0.05, ×p<0.001 and ßp<0.001 compared to 1:10; ρp<0.05 compared to 2.5:1; ‡p<0.01 compared to 4:1.

Next, we evaluated the effect of different ratios of n6/n3 FAs on the proliferation of both cancerous and non-cancerous cells by trypan blue dye exclusion method. Compared to the untreated control cells, significant reduction in cell growth was observed in all the cell lines after treatment with 1:1 ratio of n6/n3 fatty acids (Fig 4). However, low n6/n3 FA ratios selectively reduced the proliferation of only breast cancer cells (Fig 4A and 4B) and not MCF10A (Fig 4C) compared to the untreated control cells. At 1:10 ratio, there was a profound decrease in the proliferation of breast cancer cells (Fig 4A and 4B)(p<0.001). On the other hand, increasing ratios of n6/n3 induced a ratio-dependent decrease in the proliferation of both the breast cancer (Fig 4A and 4B) and non-cancerous cell lines (Fig 4C). Similar results were obtained in HaCaT and HEK293 cells wherein low ratios didn’t affect their proliferation compared to higher ratios (S2 Fig).

Thus, compared to lower ratios of n6/n3, higher ratios decreased the viability and proliferation of both the breast cancer and non-cancerous cell lines.

### Varying n6/n3 FA modulated lipid peroxidation in breast cancer cells

**Fig 5:**
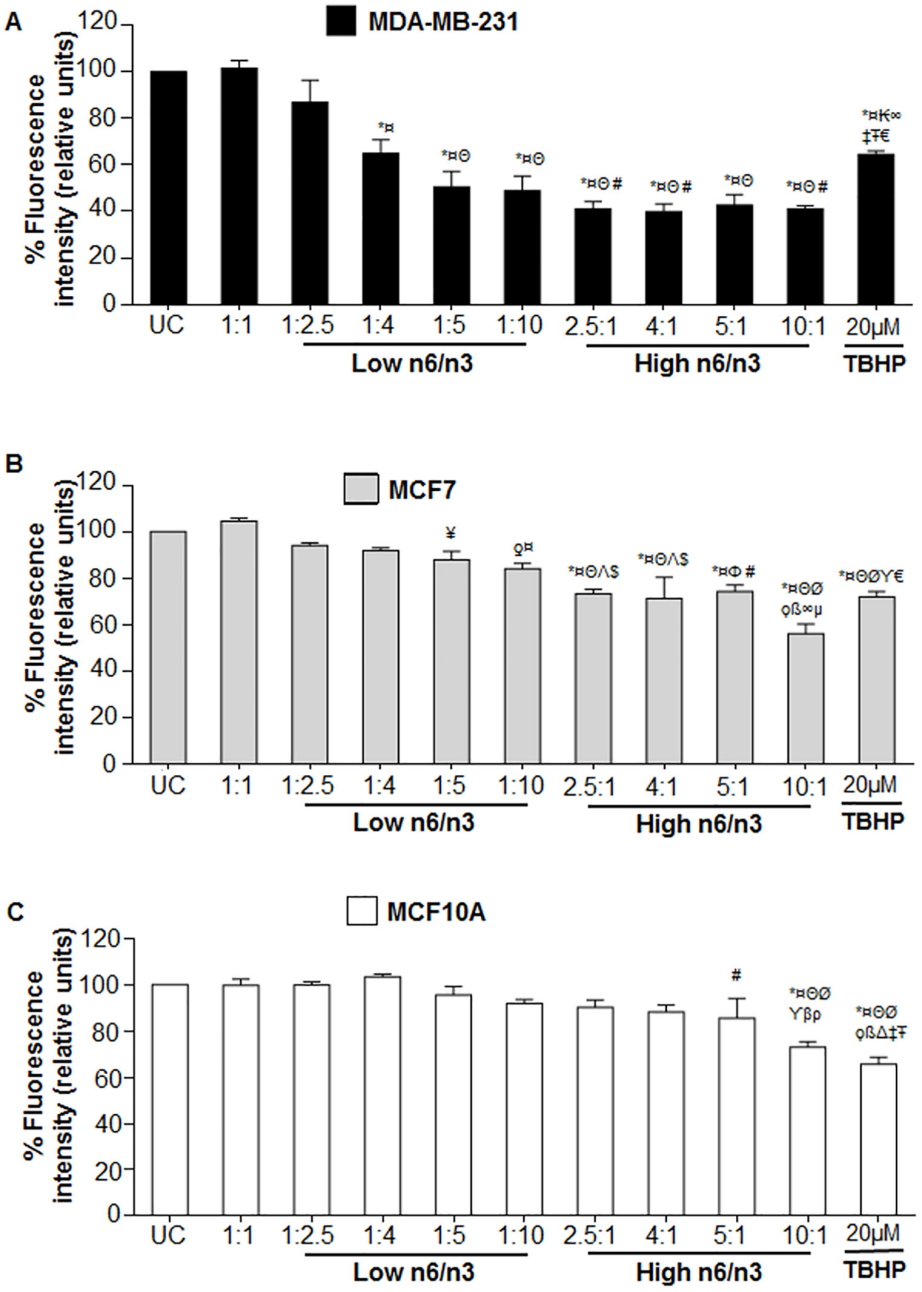
Different ratios of n6 and n3 regulate the lipid peroxidation in breast cancer and non-cancerous cells. (A) MDA-MB-231, (B) MCF7 and (C) MCF10A cells were treated with low and high n6/n3 ratios for 24h. Next day, lipid peroxidation was analyzed by using cis-parinaric acid and the values have been plotted in terms of percentage fluorescent intensity. Decrease of cis-parinaric acid fluorescence is proportional to increase in lipid peroxidation. Data has been presented as mean±SEM of three independent experiments, each conducted in triplicates. ƍp<0.01 and *p<0.001 compared to UC; ¥p<0.01 and ¤p<0.001 compared to 1:1; ₭p<0.01, Φp<0.01 and Θp<0.001 compared to 1:2.5; #p<0.05, Λp<0.01 and Øp<0.001 compared to 1:4; $p<0.05, ϒp<0.01 and ϙp<0.001 compared to 1:5; βp<0.05, ßp<0.001 as compared to 1:10; ∞p<0.01 and Δp<0.001 compared to 2.5:1; ‡p<0.01 as compared to 4:1; Ŧp<0.05 compared to 5:1; μp<0.01 compared to 5:1; €p<0.01 compared to 10:1.

It was observed that with low n6/n3 FA, there was a ratio-dependent decrease in cis-parinaric acid fluorescence intensity that is inversely proportional to lipid peroxidation levels [41]. Thus, there was increase in LPO activity in breast cancer cells compared to untreated control cells, the increase being more pronounced in MDA-MB-231 (Fig 5A) compared to MCF7 (Fig 5B). However, there was no lipid peroxidation in MCF10A (Fig 5B). Contrarily, increasing n6/n3 fatty acid ratios induced a significant increase in LPO not only in breast cancer cells (Fig 5A and 5B) but also in MCF10A (Fig 5C) compared to the untreated control cells (p<0.001). Interestingly, 1:1 ratios of n6/n3 didn’t induce any LPO in the treated cell lines. It was interesting to note that irrespective of the increasing n6/n3 ratios, the increase in LPO remained constant at ~57% to ~60% in MDA-MB-231 from 2.5/1 to 10/1 (Fig 5A); ~25% to 29% in MCF7 cells from 2.5/1 to 5/1 and 56% for 10/1 (Fig 5B). Similar trend was observed in HaCaT and HEK293 cells wherein low n6/n3 didn’t induce any LPO in the cells whereas high n6/n3 induced LPO in all the ratios (S3 Fig). Interestingly, 1:5 and 1:10 ratios of n6/n3 induced significant increase in the LPO in both MDA-MB-231 and MCF7 (p<0.001) (Fig 5A and 5B). Tert-butyl hydroperoxide (TBHP) was used as a positive control in the experiment. Thus, low n6/n3 FA ratios specifically induced LPO in breast cancer cells without affecting the non-cancerous cells.

### Omega 3 fatty acids regulated the expression of MAR binding tumor regulatory proteins

**Fig 6:**
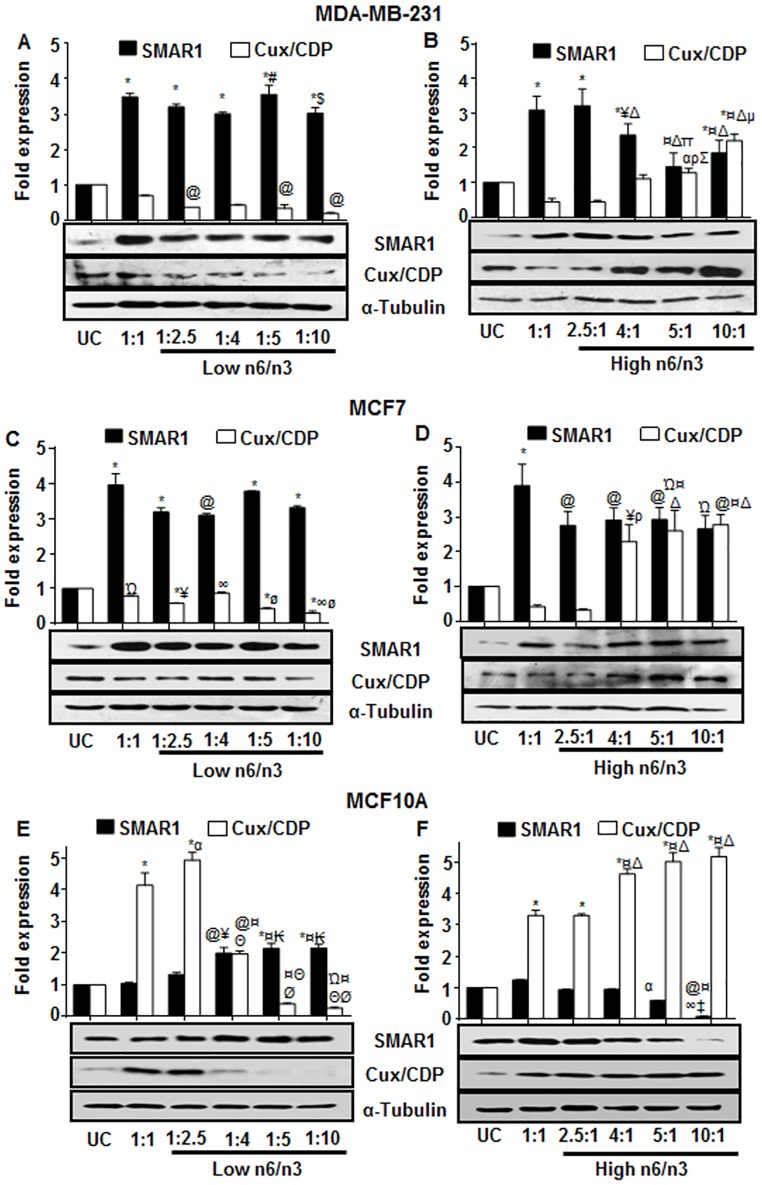
Regulation of MAR binding tumor regulatory proteins by n6/n3 ratios in breast cancer and non-cancerous cells. Expression of SMAR1 and Cux/CDP proteins was analyzed in MDA-MB-231 (A, B); MCF7 (C,D) and MCF10A (E,F). The effect of low (A, C, E) and high (B, D, F) n6/n3 FA ratios has been shown in MDAMB231, MCF7 and MCF10A cell lines, respectively. The bands were quantified by densitometry using ImageJ 1.44p (National Institutes of Health, USA, http://imagej.nih.gov/ij) and have been presented as mean±SEM of three different experiments. Ώp<0.05, @p<0.01 and *p<0.001 compared to UC; αp<0.05, ¥p<0.01 and ¤p<0.001 compared to 1:1; ₭p<0.01and Θp<0.001 compared to 1:2.5; #p<0.05 and Øp<0.001 compared to 1:4; $p<0.05 compared to 1:5;ρp<0.05, ∞p<0.01 and Δp<0.001 compared to 2.5:1; ‡p<0.01 and Σp<0.001 compared to 4:1, μp<0.01 and πp<0.001 compared to 5:1.

We wanted to evaluate the effect of varying n6/n3 ratios on the expression of MAR binding proteins since they are recently considered as primary diagnostic and/or prognostic markers [43]. We found that in both MDA-MB-231 and MCF7, all the ratios of n3 and n6 FAs significantly increased the expression of tumor suppressor MARBP SMAR1, compared to the untreated control cells (Fig 6A–6D). The expression of SMAR1 was significantly increased in MDA-MB-231 treated with low n6/n3 ratios (Fig 6A) compared to those treated with high n6/n3 (Fig 6B) (p<0.05). In MCF7, SMAR1 expression was increased after treatment with low n6/n3; however with high n6/n3, expression of SMAR1 was not significantly reduced (p = 0.069) (Fig 6C and 6D). Interestingly, there was a significant decrease in the expression of tumor activator MARBP Cux/CDP in low n6/n3 FA ratios in both MDA-MB-231 and MCF7, compared to the untreated control cells (Fig 6A and 6C, respectively) (p<0.05). On the other hand, the higher ratios of n6/n3 (from 4/1 upto 10/1) were found to significantly increase the expression of Cux/CDP in both MDA-MB-231 and MCF7 (Fig 6B and 6D, respectively) compared to the untreated control cells (p<0.05). Among all the tested n6/n3 ratios, 1:10 ratio showed profound decrease in Cux/CDP expression in both the breast cancer cell lines.

In MCF10A, compared to the untreated control cells, SMAR1 expression was increased in all the ratios of low n6/n3, the increase being more significant in 1:4, 1:5 and 1:10 ratios (Fig 6E). However, high n6/n3 FAs, particularly, 5:1 and 10:1 ratios, decreased the expression of SMAR1 significantly (Fig 6F). Surprisingly, 1:1 and 1:2.5 of low n6/n3 showed increase in the expression of Cux/CDP that was later decreased in 1:4, 1:5 and 1:10 n6/n3 ratios (Fig 6E). On the contrary, high n6/n3 increased the expression of Cux/CDP more significantly at 4:1, 5:1 and 10:1 ratios (Fig 6F).

### Differential ratios of n6/n3 regulate p21^WAF1/CIP1^ expression in breast cancer cells

**Fig 7:**
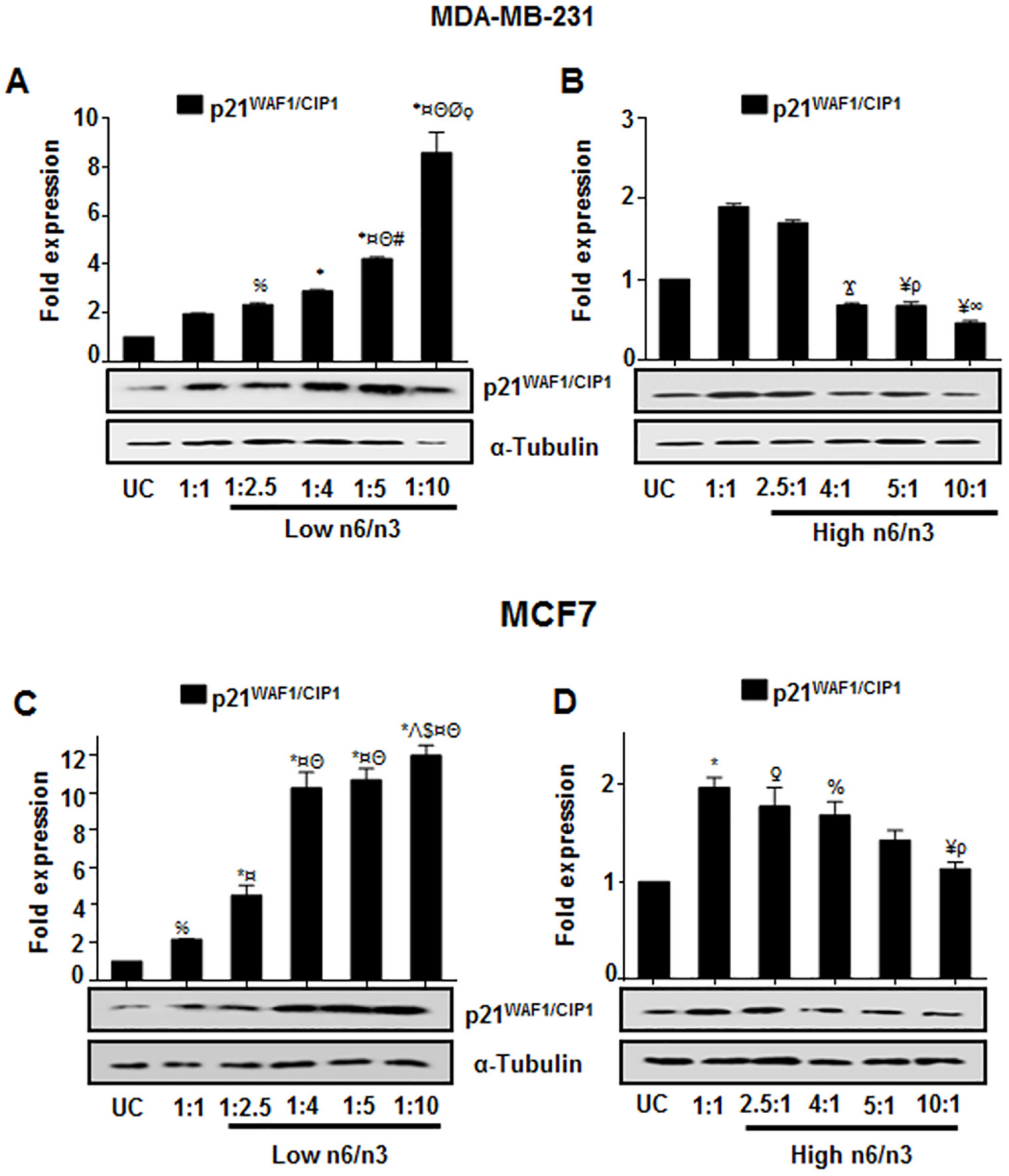
Differential ratios of n6/n3 regulate p21^WAF1/CIP1^ expression in breast cancer cells. Expression of p21^WAF1/CIP1^ protein was analyzed in MDA-MB-231 (A, B) and MCF7 (C, D) that show the effect of low (A, C) and high (B, D) n6/n3 FA ratios. The bands were quantified by densitometry using ImageJ 1.44p (National Institutes of Health, USA, http://imagej.nih.gov/ij) and have been presented as mean±SEM of three different experiments. %p<0.05, ƍp<0.01 and *p<0.001 compared to UC; Ϫp<0.05, ¥p<0.01 and ¤p<0.001 compared to 1:1; Θp<0.001 compared to 1:2.5; #p<0.05 compared to 1:4; Λp<0.01 compared to 1:4; Øp<0.001as compared to 1:4; $p<0.05 as compared to 1:5; ρp<0.05 and ∞p<0.01 compared to 2.5:1.

It was found that there was a significant increase in p21^WAF1/CIP1^ expression in MDA-MB-231 (Fig 7A) and MCF7 (Fig 7C) cells treated with low n6/n3 FAs; the increase being ratio dependent in MDA-MB-231. However, expression of p21^WAF1/CIP1^ decreased with increasing ratios of n6/n3 fatty acids (Fig 7B for MDA-MB-231 and Fig 7D for MCF7). Thus activation of SMAR1, by n3 fatty acids, lead to the activation of p21^WAF1/CIP1^ that could regulate the cell growth.

### Different n6/n3 FAs induced changes in fatty acid composition of the breast cancer cells

**Fig 8:**
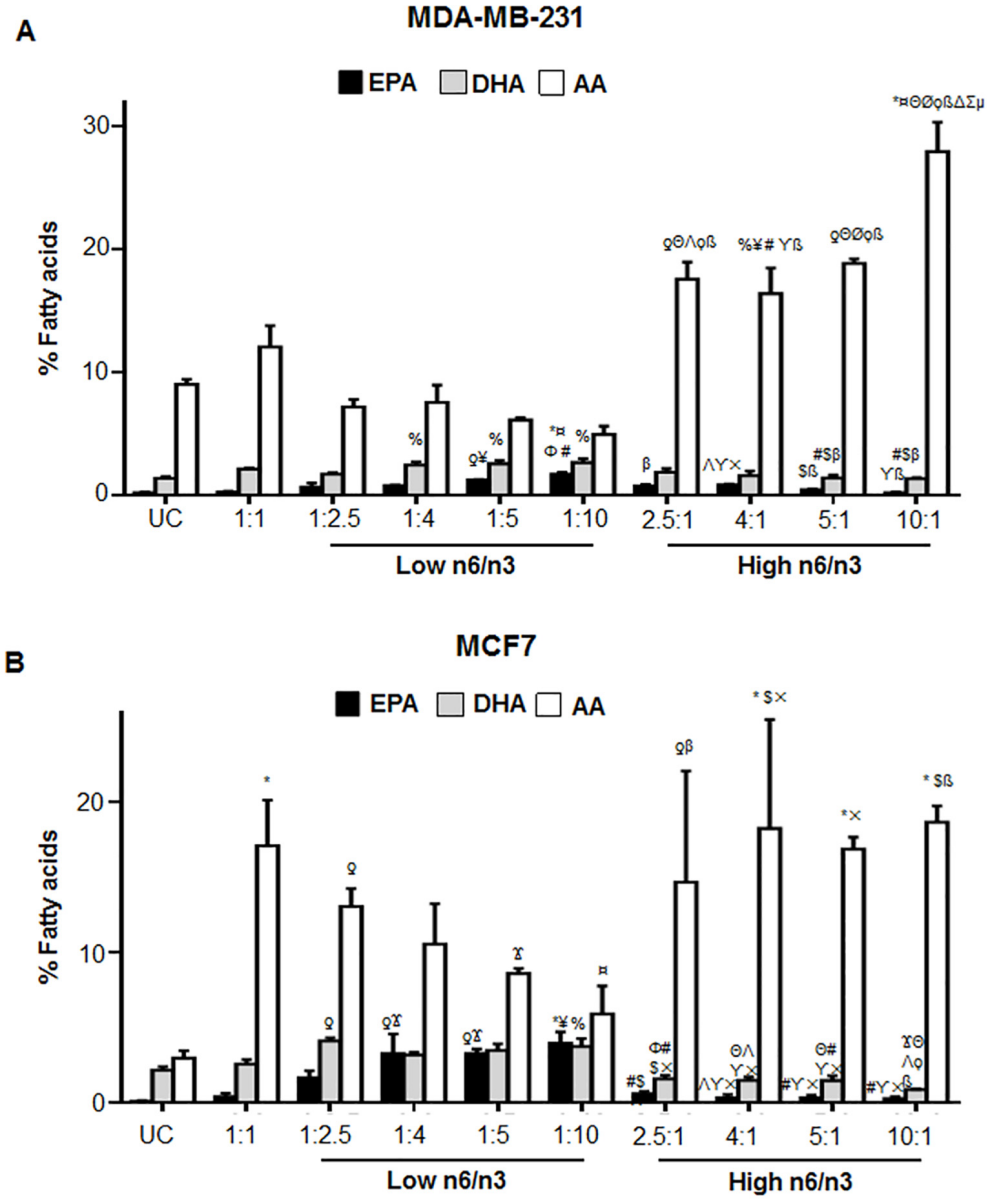
Relative percentage of EPA, DHA and AA in breast cancer cell lines. The cells were treated with different ratios of n6 and n3 FA for 24h. The levels of EPA, DHA and AA has been shown in MDA-MB-231 (A) and MCF7 (B) cells treated with low and high n6/n3 ratios. Each value represents mean±SEM of three independent experiments. %p<0.05, ƍp<0.01 and *p<0.001 compared to UC; Ϫp<0.05, ¥p<0.01 and ¤p<0.001 compared to 1:1; Φp<0.01 and Θp<0.001 compared to 1:2.5; #p<0.05, Λp<0.01 and Øp<0.001 compared to 1:4; $p<0.05, ϒp<0.01 and ϙp<0.001 compared to 1:5; βp<0.05, ×p<0.001 and ßp<0.001 compared to 1:10; Δp<0.001 compared to 2.5:1; Σp<0.001 compared to 4:1; μp<0.01 compared to 5:1.

To detect whether treatment with different ratios of n6 and n3 fatty acids induced any specific changes in the fatty acid composition of the cells, we analyzed the fatty acid profile of the total cell lipid extract of the samples. Fig 8 shows the relative fatty acid percentage of EPA, DHA and AA in the lipid fractions of MDA-MB-231 and MCF7 after treatment with different n6/n3 FA.

Both the cell lines showed efficient incorporation of respective fatty acids (EPA, DHA and AA) since supplementation of each fatty acid increased its own level in MDA-MB-231 and MCF7 cells (Fig 8). In MDA-MB-231, compared to untreated control cells, there was an increase in EPA and DHA content in low n6/n3 FA ratios, which decreased with increasing ratios (Fig 8A). Interestingly, there was a reduction of AA in presence of low n6/n3 FA, which increased in higher ratios (Fig 8A) compared to both untreated control cells as well as 1/1 ratio of n6/n3.

Similarly, in MCF7 cells, compared to untreated control cells, there was an increase in EPA and DHA content at low n6/n3 with a concomitant decrease at higher ratios (Fig 8B). An interesting observation was that MCF7 cells showed high AA in all the FA ratios compared to the untreated control cells (Fig 8B) as reported earlier [44]. However, compared to 1/1 ratio, with lower n6/n3 ratios, there was a decrease in AA and non-significant increase in higher ratios (Fig 8B). Nevertheless, compared to the untreated control cells, there was a significant increase in AA levels.

**Fig 9:**
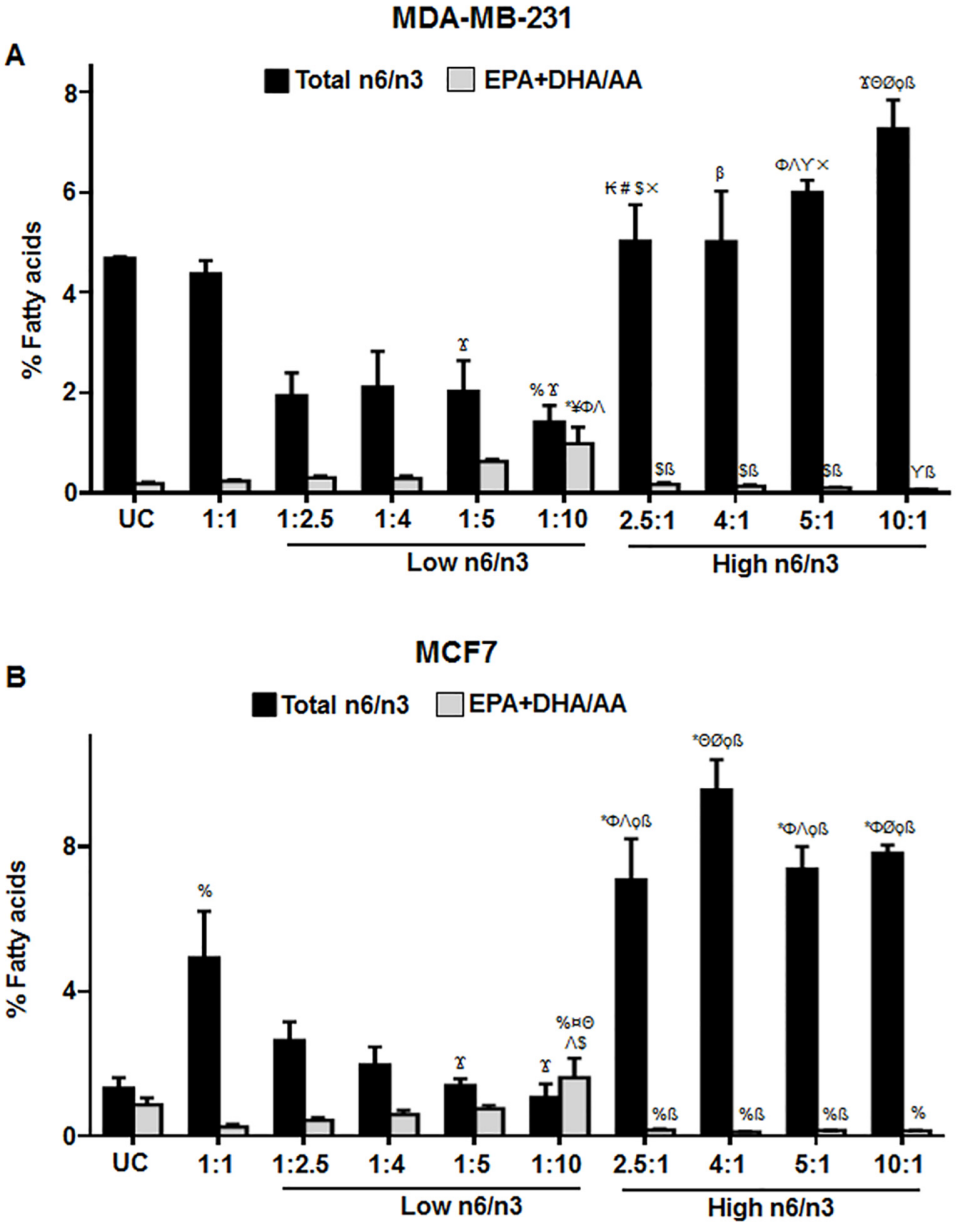
Relative percentage of total n6/n3 and EPA+DHA/AA ratio in breast cancer cells. The cells were treated with different ratios of n6 and n3 FA for 24h. The levels of total n6/n3 and EPA+DHA/AA has been shown in MDA-MB-231 (A) and MCF7 (B) cells treated with low and high n6/n3 ratios, respectively. Total n6 fatty acids include Linoleic acid (LA) (18:2n6), Gamma-linolenic acid (GLA) (18:3n6), Dihomo-gamma-linolenic acid (DGLA) (20:3n6), Arachidonic acid (AA) (20:4n6); Total n3 fatty acids include ALA (18:3n3), EPA (20:5n3), DHA (22:6n3), DPA(22:5n3). Each value represents mean±SEM of three independent experiments. %p<0.05 and *p<0.001 compared to UC; Ϫp<0.05, ¥p<0.01 and ¤p<0.001 compared to 1:1; ₭p<0.01, Φp<0.01 and Θp<0.001 compared to 1:2.5; #p<0.05, Λp<0.01 and Øp<0.001 compared to 1:4; $p<0.05, ϒp<0.01 and ϙp<0.001 compared to 1:5; βp<0.05, ×p<0.001 and ßp<0.001 compared to 1:10.

We further analyzed the effect of different ratios on the total n6/n3 FA and EPA+DHA/AA ratios in both MDA-MB-231 and MCF7 (Fig 9). In MDA-MB-231, the untreated control cells as well as those treated with 1:1 ratio of n6/n3 showed a high content of total n6/n3 FA (Fig 9A). However, when cells were treated with low ratios of n6/n3, there was a significant decrease in total n6/n3 that remained almost constant in 1:2.5, 1:4 and 1:5 ratios with more decrease at 1:10 ratio (Fig 9A). In MCF7, the untreated control cells didn’t have high total n6/n3 (Fig 9B). However, at 1:1 ratio, there was a significant increase in total n6/n3 that decreased with lower ratios of n6/n3 (Fig 9B). At higher n6/n3 ratios, there was increase in total n6/n3 in both MDA-MB-231 and MCF7 (Fig 9A and 9B).

In MDA-MB-231, EPA+DHA/AA was found to increase at low n6/n3 compared to untreated control cells or 1:1 ratio, the increase being more at 1:10 (Fig 9A). In MCF7, EPA+DHA/AA was found to increase compared to 1:1 ratio, Interestingly, the untreated control cells had more EPA+DHA/AA that decreased at 1:1 ratio, but retained its original level at 1:5 ratio of low n6/n3, and increased significantly at 1:10 ratio (Fig 9B). At high n6/n3, EPA+DHA/AA decreased significantly compared to untreated control and 1:1 ratios (Fig 9A and 9B).

Among all the ratios tested, 1:10 ratio of low n6/n3 showed significant increase in EPA and EPA+DHA/AA compared to untreated control and 1:1 ratio in both the breast cancer cell lines. On the other hand, compared to 1:1 ratio of low n6/n3, AA and total n6/n3 were significantly decreased at 1:10 ratio.

### Discussion

Various sources of information suggest that human beings have evolved on a diet with a ratio of omega-6 to omega-3 essential fatty acids (EFA) of 1 [32]. However, nowadays, the ratio has drastically increased due to change in dietary pattern having high propensity towards westernized diet that has n6/n3 ratio of 15/1–16.7/1 [45, 46]. Thus, in the present study, for the first time high and low ratios of n6/n3 FAs were used to mimic diets of different populations [32, 47] in vitro to evaluate their effect on cellular responses. Equal ratio (1:1) of n6/n3 was chosen to mimic the ancestral diet; 4:1 and 5:1 ratios were chosen to mimic Japanese and Indian rural diet, respectively; 10:1 was chosen to mimic US, UK and European dietary pattern that showed excessive intake (between 15–16) of n6 FAs [32, 47]. 1:2.5 ratio of n6/n3 FAs was chosen based upon previous research in animal models [35, 36, 48, 49]. Moreover, our common diet generally consist of varying levels of n3 and n6 fatty acids, and thus, it becomes essential to systematically analyze the effects of varying ratios of these fatty acids on growth regulation of breast cancer cells. In the current study, we found that low ratios of n6/n3 fatty acids preferentially killed the breast cancer cells; modulated their lipid peroxidation and critically controlled the expression of tumor regulatory MARBPs.

Low n6/n3 FAs were preferentially cytotoxic to the breast cancer cells and not to the non-cancerous cells. Contrarily, higher ratios affected the viability of all the cell lines similar to that of chemotherapeutic drugs that can not differentiate between normal and cancerous cells. Various studies have reported that PUFAs such as AA, GLA, EPA and DHA are differentially metabolized by normal and tumor cells as well as drug-sensitive and drug resistant cells [50–54]. This explains as to how different ratios of n6/n3 exhibited differential effect on cell viability of different cell lines. The normal cells metabolize PUFAs to produce cytoprotective lipids such as lipoxins, resolvins and protectins whereas cancerous cells generate toxic hydroperoxy fatty acids [50, 51]. Earlier studies have reported unesterified arachidonic acid as a signal for induction of apoptosis in cells. Exogenous AA has been shown to induce apoptosis in colon cancer and other cell lines including HEK 293 [55]. This could be one of the reasons as to how high n6/n3 ratios could affect the viability and proliferation of all the cell types compared to the lower ratios.

We have previously reported that ALA increased lipid peroxidation in both the breast and cervical cancer cell lines with a simultaneous decrease in cell proliferation [8]. A similar trend was observed in the breast cancer cell types treated with different n6/n3 ratios. The decrease in cell proliferation was found to positively correlate with the increase in lipid peroxidation levels. AA has been reported to generate more thiobarbituric acid-reactive material (TBARM) [56] thereby inducing more cytotoxicity compared to DHA. Interestingly, MCF7 cells (ER/PR positive), showed less lipid peroxidation compared to MDA-MB-231 (ER/PR negative), in response to fatty acid treatment. The reason could be that estrogen is known to increase the resistance towards ROS generation [57, 58]. Recently, it was shown that supplementation of 1/1 ratio of fish/corn oil in rats, induced with colon cancer, decreased ROS, thioredoxin reductase (TrxR) and apoptosis with a concomitant increase in the antioxidant activity [48] in the initiation phase. The absence of LPO in cells treated with 1/1 ratio of n6/n3 FAs, observed in the current report, could be probably due to generation of antioxidants that neutralized the free radicals. The cytotoxic action of omega 3 fatty acids seems to depend on their ability to increase free radical generation and lipid peroxidation [50, 52, 59] that damage a variety of enzymes, proteins and DNA, thereby, leading to cell death. Thus, a right balance between n6 and n3 fatty acids is highly important in regulating the tumor growth.

Both the breast cancer cell lines showed a significant increase in total n3 FAs with a corresponding decrease in total n6 FAs in low n6/n3 compared to higher ratios. The ratio of EPA+DHA/AA also increased in low n6/n3 FA and decreased in higher ratios in both the cell types. Interestingly, MCF7 showed higher percentage of AA compared to that of MDA-MB-231. Estrogens have been known to promote the metabolic conversion of PUFAs more rapidly and thus synthesis of AA and DHA from their precursors may be enhanced through an ER-dependent pathway [60, 61]. Moreover, treatment of the cells with n3 PUFAs decreased the levels of AA more in MDA-MB-231 than in MCF7, which has been reported earlier [44]. High fat diet (primarily in the form of n6 PUFAs) has been known to be one of the factors that elevates estrogen (E2) levels and its circulating levels during pregnancy has been reported to increase the risk of developing breast cancer [62].

In the present report, we are for the first time showing that the omega fatty acids modulate the expression of MARBPs such as SMAR1 and Cux/CDP. MARBPs help in attachment of MARs, AT-rich DNA sequences, to the nuclear matrix (NM) resulting into organization of genomic DNA into topologically independent loop domains that are implicated in transcription, replication, repair, recombination, demethylation and chromatin accessibility [63, 64]. Changes in chromatin structure could modulate gene expression resulting into genomic instability that may lead to cellular transformation and malignant outgrowth. Thus, MARBPs that control chromatin organization play key role in cancer progression. Aberrant expression of MARBPs such as PARP, CUTL1, HMG (I/Y), RUNX1-3, SATB1, SATB2, PcG (polycomb group of proteins), SAFB1/2, SMAR1 etc. has been implicated in several cancers [65–67]. SMAR1, a tumor suppressor MARBP, is down regulated in majority of cancers including breast cancer [64, 68] as well as in MCF7 and MDA-MB-231 [69]. Cux/CDP/CUTL1/Cux-1 is another MARBP that is significantly increased in high-grade carcinomas and its expression is inversely correlated with breast cancer survival [70]. Cux/CDP has been shown to enhance tumor cell invasion and migration, besides causing malignancies in several organs and cell types [70, 71]. After treatment of the breast cancer cells with the low n6/n3, there was an enhanced expression of SMAR1 with a simultaneous decrease in the expression of Cux/CDP. Interestingly, the endogenous level of Cux/CDP was quite high in untreated control cells that were reduced after treatment with low n6/n3 ratios. The expression of SMAR1 has been reported to be inversely correlated with that of Cux/CDP in breast cancer [64]. It was interesting to note that MCF10A cells showed increased expression of SMAR1 when treated with low n6/n3 ratios and decreased expression in presence of high ratios. Moreover, Cux/CDP was decreased in presence of low n6/n3 ratios and increased in higher ratios. Surprisingly, 1:1 and 1:2.5 of low n6/n3 FAs increased the expression of Cux/CDP that was later decreased in cells treated with 1:4, 1:5 and 1:10 n6/n3 ratios (Fig 6E). The up-regulated Cux/CDP expression could be justified by its role in organ development such as brain, limb, lung, kidney, cell differentiation, adhesion, motility and invasiveness [72, 73]. A recent report has shown that ectopic expression of CDP/Cux in MCF10A cells stimulated cell migration and invasion capacity compared to the non-expressing cells [74]. These results suggest that if non-cancerous cells are exposed to higher concentrations of n6 fatty acids, they may tend to acquire cancerous phenotype through deregulation of tumor marker proteins. Thus, regulation of MARBPs by omega 3 fatty acids could be a potential therapeutic target for regulation of cancer growth.

It is known that SMAR1 activates p53 and induces p21 expression that in turn regulates G_1_ or G_2_ checkpoints of the cell cycle [63]. In this context, we found that in untreated breast cancer cells, SMAR1 and p21^WAF1/CIP1^ expressions were highly reduced compared to that of Cux/CDP. However, low n6/n3 FAs not only induced expression of SMAR1 but also activated cell cycle regulatory protein, p21^WAF1/CIP1^, which in turn regulated breast cancer growth. Contrarily, high n6/n3 ratios not only increased CDP/Cux but also decreased SMAR1 and p21^WAF1/CIP1^ expressions, thereby promoting breast cancer growth.

Our results show that differential ratios of n6/n3 fatty acids could significantly affect the growth kinetics of breast cancer cells and regulate chromatin modulatory proteins. Even though the ratio of 1:10 of n6/n3 FAs show promising results, the overall data indicates that even minimal increase in n3 FAs in our diet could regulate cellular machinery resulting into improved health.

## Conclusion

Varying ratios of n6 and n3 fatty acids in the diet can modulate the intrinsic signal transduction mechanisms that in turn can regulate the cell growth. Even though it is quite impossible to revert back to our ancestral diet, however, efforts can be made to reduce the ratio of n6/n3 fatty acids in our diet. By modifying the fatty acid composition of the cells, many aspects of cancer cell metabolism could be regulated. Thus, the risk of cancer would be reduced by restricting the intake of n6 fatty acids in our diet.

## Supporting Information

S1 Fig Differential ratios of n6and n3fatty acids regulate the viability of non-cancerous cells. HaCaT and HEK293 cells were treated with different ratios of n6 (AA) and n3 (EPA+DHA) fatty acids and analyzed for viability by MTT assay. Data has been presented as mean±SEM of three independent experiments, each conducted in triplicates. %p<0.05, ƍp<0.01 and *p<0.001 compared to UC; ¥p<0.01 and ¤p<0.001 compared to 1:1; Φp<0.01 and Θp<0.001 compared to 1:2.5; Λp<0.01 and Øp<0.001 compared to 1:4; ϒp<0.01 and ϙp<0.001 compared to 1:5; βp<0.05, ×p<0.001 and ßp<0.001 compared to 1:10.
(TIF)

S2 Fig Differential ratios of n6and n3regulate the cell proliferation of non-cancerous cell lines. HaCaT and HEK293 cells were treated with different ratios of n6 (AA) and n3 (EPA+DHA) FA for 24h. Next day, the number of viable cells was counted using trypan blue dye exclusion assay. Data has been presented as mean±SEM of three independent experiments, each conducted in triplicates. ƍp<0.01 and *p<0.001 compared to UC; ¤p<0.001 compared to 1:1; Φp<0.01 and Θp<0.001 compared to 1:2.5; Λp<0.01 and Øp<0.001 compared to 1:4; ϒp<0.01 and ϙp<0.001 compared to 1:5; ×p<0.001 and ßp<0.001 compared to 1:10; ρp<0.05 and Δp<0.001 compared to 2.5:1; ‡p<0.01 and Σp<0.001 compared to 4:1, μp<0.01 compared to 5:1.
(TIF)

S3 Fig Differential ratios of n6and n3regulate the lipid peroxidation in non-cancerous cells. HaCaT and HEK293 cells were treated with different ratios of n6 (AA) and n3 (EPA+DHA) ratios for 24h. Next day, lipid peroxidation was analyzed by using cis-parinaric acid and the values have been plotted in terms of percentage fluorescent intensity. Decrease of cis-parinaric acid fluorescence is proportional to increase in lipid peroxidation. Data has been presented as mean±SEM of three independent experiments, each conducted in triplicates. %p<0.05, ƍp<0.01 and *p<0.001 compared to UC; ¥p<0.01 compared to 1:1; ₭p<0.01, Φp<0.01 and Θp<0.001 compared to 1:2.5; Λp<0.01 and Øp<0.001 compared to 1:4; $p<0.05, ϒp<0.01 and ϙp<0.001 compared to 1:5; βp<0.05, ×p<0.001 and ßp<0.001 compared to 1:10.
(TIF)
