# Supplementary figures and images for: Differential Ratios of Omega Fatty Acids (AA/EPA+DHA) Modulate Growth, Lipid Peroxidation and Expression of Tumor Regulatory MARBPs in Breast Cancer Cell Lines MCF7 and MDA-MB-231
Source: PLoS One. 2015 Sep 1;10(9):e0136542. doi: 10.1371/journal.pone.0136542 (PMC4556657; doi:10.1371/journal.pone.0136542)

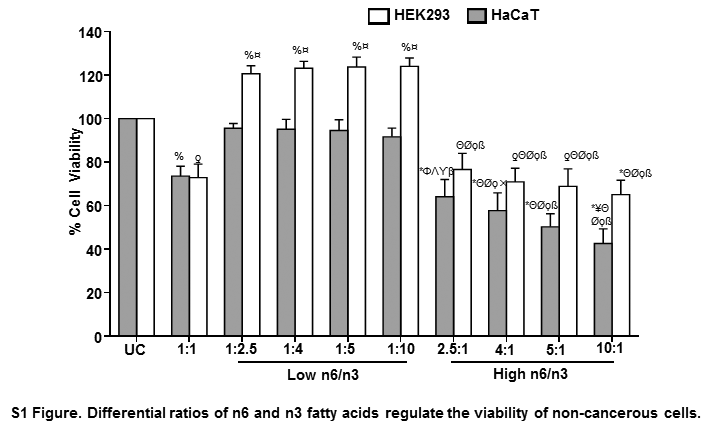

Supplement: S1 Fig — HaCaT and HEK293 cells were treated with different ratios of n6 (AA) and n3 (EPA+DHA) fatty acids and analyzed for viability by MTT assay. Data has been presented as mean±SEM of three independent experiments, each conducted in triplicates. %p<0.05, ƍp<0.01 and *p<0.001 compared to UC; ¥p<0.01 and ¤p<0.001 compared to 1:1; Φp<0.01 and Θp<0.001 compared to 1:2.5; Λp<0.01 and Øp<0.001 compared to 1:4; ϒp<0.01 and ϙp<0.001 compared to 1:5; βp<0.05, ×p<0.001 and ßp<0.001 compared to 1:10. (TIF) [file pone.0136542.s001.tif]

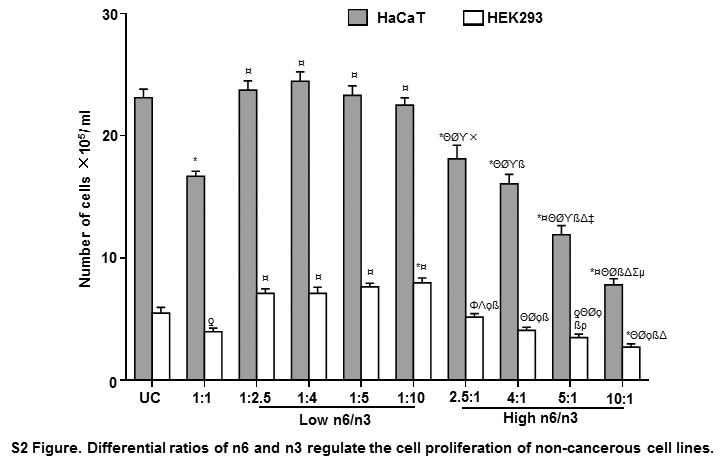

Supplement: S2 Fig — HaCaT and HEK293 cells were treated with different ratios of n6 (AA) and n3 (EPA+DHA) FA for 24h. Next day, the number of viable cells was counted using trypan blue dye exclusion assay. Data has been presented as mean±SEM of three independent experiments, each conducted in triplicates. ƍp<0.01 and *p<0.001 compared to UC; ¤p<0.001 compared to 1:1; Φp<0.01 and Θp<0.001 compared to 1:2.5; Λp<0.01 and Øp<0.001 compared to 1:4; ϒp<0.01 and ϙp<0.001 compared to 1:5; ×p<0.001 and ßp<0.001 compared to 1:10; ρp<0.05 and Δp<0.001 compared to 2.5:1; ‡p<0.01 and Σp<0.001 compared to 4:1, μp<0.01 compared to 5:1. (TIF) [file pone.0136542.s002.tif]

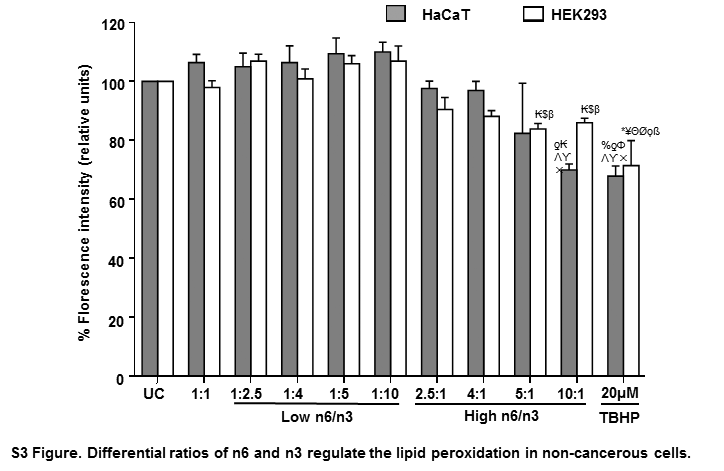

Supplement: S3 Fig — HaCaT and HEK293 cells were treated with different ratios of n6 (AA) and n3 (EPA+DHA) ratios for 24h. Next day, lipid peroxidation was analyzed by using cis-parinaric acid and the values have been plotted in terms of percentage fluorescent intensity. Decrease of cis-parinaric acid fluorescence is proportional to increase in lipid peroxidation. Data has been presented as mean±SEM of three independent experiments, each conducted in triplicates. %p<0.05, ƍp<0.01 and *p<0.001 compared to UC; ¥p<0.01 compared to 1:1; ₭p<0.01, Φp<0.01 and Θp<0.001 compared to 1:2.5; Λp<0.01 and Øp<0.001 compared to 1:4; $p<0.05, ϒp<0.01 and ϙp<0.001 compared to 1:5; βp<0.05, ×p<0.001 and ßp<0.001 compared to 1:10. (TIF) [file pone.0136542.s003.tif]
